# Transcriptome sequencing analysis of alfalfa reveals CBF genes potentially playing important roles in response to freezing stress

**DOI:** 10.1590/1678-4685-GMB-2017-0053

**Published:** 2017-11-06

**Authors:** Yongjun Shu, Wei Li, Jinyue Zhao, Sijia Zhang, Hanyun Xu, Ying Liu, Changhong Guo

**Affiliations:** 1 College of Life Science and Technology, Harbin Normal University, Harbin Heilongjiang, China

**Keywords:** *Medicago sativa* L., RNA-seq, freezing tolerance, C-repeat binding factors (CBF)

## Abstract

Alfalfa (*Medicago sativa* L.) is an important perennial forage, with high nutritional value, which is widely grown in the world. Because of low freezing tolerance, its distribution and production are threatened and limited by winter weather. To understand the complex regulation mechanisms of freezing tolerance in alfalfa, we performed transcriptome sequencing analysis under cold (4 °C) and freezing (-8 °C) stresses. More than 66 million reads were generated, and we identified 5767 transcripts differentially expressed in response to cold and/or freezing stresses. These results showed that these genes were mainly classified as response to stress, transcription regulation, hormone signaling pathway, antioxidant, nodule morphogenesis, etc., implying their important roles in response to cold and freezing stresses. Furthermore, nine CBF transcripts differentially expressed were homologous to CBF genes of Mt-FTQTL6 site, conferring freezing tolerance in *M. truncatula*, which indicated that a genetic mechanism controlling freezing tolerance was conservative between *M. truncatula* and *M. sativa*. In summary, this transcriptome dataset highlighted the gene regulation response to cold and/or freezing stresses in alfalfa, which provides a valuable resource for future identification and functional analysis of candidate genes in determining freezing tolerance.

## Introduction

In general, plants are always challenged by various unfavorable environmental conditions, such as low temperatures, water deficit, and salinity. Among these stresses, low temperatures are one of the major factors, limiting plant growth, development, and distribution. In order to adapt to low temperature conditions, plants have employed numerous regulation mechanisms to survive through cold and/or freezing stresses (Knight and Knight, 2012). Recently, important progress have revealed that prior exposure to non-freezing low temperatures improved freezing tolerance of a plant, which is known as cold acclimation (Thomashow, 1999). In plant cold acclimation process, there is a wide range of physiological, biochemical, metabolic and gene expression altering. In plants, including Arabidopsis, the molecular regulation mechanisms of cold acclimation and acquired freezing tolerance have been extensively investigated. Many reports have showed that C-repeat (CRT) binding factors (CBF), also known as dehydration responsive elements binding factors (DREB), have played fundamental roles in plant adaption to low temperature, prominently determining plant freezing tolerance (Thomashow, 2010). CBF genes are rapidly induced by low temperature stress, activating the expression of many downstream genes, known as CBF regulon (Gilmour *et al.*, 2004). In addition, transcriptions of CBF genes are also regulated by other functional genes, for example, MYB transcription factor, ICE1 and ICE2; these genes constitute a complex regulation pathway response to low temperature stress (Zarka *et al.*, 2003; Kim *et al.*, 2015). In plant genomes, CBF genes are always physically linked in tandem cluster, and they encode closely related proteins responses to cold stress. For example, in Arabidopsis, three CBF genes, which are induced by low temperature condition (Lin *et al.*, 2008), are clustered within an 8.7-kb region in chromosome four. In *Medicago truncatula*, ten CBF genes (MtERF34–43) have clustered within an approximately 393-Kb region on chromosome six, reported as Mt-FTQTL6 site, conferring freezing tolerance (Tayeh *et al.*, 2013); six of them were identified to respond to cold and/or freezing stress by transcriptome sequencing (Shu *et al.*, 2015).

Alfalfa (*Medicago sativa* L.) is an important perennial forage legume species with high nutritional value, widely distributed in temperate zones of the world, including US, Canada, and China. However, lack of tolerance to freezing stress has limited its survival, productivity, and ecological distribution, especially in high-latitude regions (Castonguay *et al.*, 2013). Little is known about freezing tolerance of alfalfa, which is a complex trait determined by numerous factors from alfalfa and the environment. Early research has revealed high correlations between fall growth and freezing tolerance, and the fall dormancy has been a useful predictor of potential freezing tolerance in alfalfa breeding process (Schwab *et al.*, 1996). However, scientists have recently found that alfalfa genotypes with non-dormant fall growth possessed modest levels of freezing tolerance, implying that the genetic regulation mechanisms of fall dormancy and freezing tolerance were different in alfalfa (Brummer *et al.*, 2000). Up to now, some functional genes have been isolated and characterized to respond to cold and/or freezing stress in alfalfa, such as dehydrin (Dube *et al.*, 2013), glycine-rich proteins (Ferullo *et al.*, 1997), heat shock transcription factors (Friedberg *et al.*, 2006), deduced polypeptide (Monroy *et al.*, 1993), etc. However, these investigations were incomplete, limiting the exploration of molecular mechanisms of freezing tolerance in detail. Recently, studies reported transcriptome analyses of gene response to cold and/or freezing stresses performed in *M. truncatula* and *M. falcata*, two plant species closely related to alfalfa, and many functional gene responses to cold stress have been well characterized (Pennycooke *et al.*, 2008; Zhang *et al.*, 2011; Miao *et al.*, 2015). These findings have provided new insights into the biochemical and molecular mechanisms involved in cold adaptation, which could be used for alfalfa genetic breeding process. Due to the complex genome in alfalfa, its genomics research is slow (Julier *et al.*, 2003). Therefore, identification and characterization of functional genes is an urgent challenge in alfalfa genetic breeding work.

Recently, the development of high-throughput sequencing technologies has provided a potentially valuable strategy for genome-wide transcriptome analysis, termed as RNA-seq (Wang *et al.*, 2009). RNA-seq provides precise measurement of levels of transcripts and their sequence information at the same time. It is highly efficient, more reliable and more cost-effective than hybridization-based microarrays, which makes it widely used to characterize the transcriptomes of plants, particularly those of non-model plant that lack genome sequences. Over the last decade, RNA-seq has been increasingly applied to investigate various plant processes, including plant development and response to stress, which have confirmed their function as powerful tools for plant genetics research (Strickler *et al.*, 2012).

In the present study, transcriptome sequencing analyses of alfalfa response to cold and freezing stresses were performed, and numerous genes responsive to cold and freezing stresses, including transcription factors, hormone signaling, antioxidant, etc, were assessed. Dozens of CBF transcripts matching to CBF cluster in *M. truncatula* were also identified as induced by cold and freezing stress, implying their critical roles in determining freezing tolerance in alfalfa.

## Materials and Methods

### Plant material and growth conditions

*M. sativa* (cv. *Zhaodong*) was domesticated and bred by Heilongjiang Animal Science Institute, Heilongjiang province, China. Alfalfa is well grown in northeastern China, with high freezing tolerance. As previously described (Shu *et al.*, 2015, 2016), the experiment of alfalfa response to cold and/or freezing stresses was performed as follow: alfalfa seeds were germinated on filter paper, and transferred to pots with mixture of perlite and sand at 3:1 in volume. The seedlings were grown in chamber, with a 14 h light period, 18 °C/24 °C (light/dark) temperature conditions, and were irrigated with 1/2 Hoagland solution every two days. After eight weeks, the alfalfa plants were randomly divided into three groups, the control group (A group) continued to grow as described above, the cold group (B group) was transferred to a new chamber set at 4 °C, and the freezing group (C group) was transferred to a chamber set at -8 °C. Five seedlings from each group were harvested at 3 h after stress treatments, and were bulked into one sample separately. All samples were frozen and stored in liquid nitrogen for RNA-seq and qRT-PCR detection.

### Construction and sequencing of alfalfa RNA-seq library

Total RNA was extracted using the RNeasy Plant Mini Kit (Qiagen, Valencia, CA, USA) following the manufacturer’s instructions. RNA samples from the same group were mixed and used to construct pair-end libraries. RNA-seq was performed on the Illumina GAII platform according to the manufacturer’s instructions to generate 100 bp pair-end reads (BGI-Shenzhen Co. Ltd, Shenzhen, China).

### Sequences assembly and annotation of alfalfa RNA-seq

Adapter sequences and low quality reads were first removed from raw sequences to produce clean data. Then, clean reads were merged and assembled *de novo* into contigs using Trinity with default parameters (Haas *et al.*, 2013). The contigs were further clustered into unigenes using iAssembler (Zheng *et al.*, 2011) and CD-HIT-EST (Li and Godzik, 2006). To evaluate their genetic information, these assembled unigenes were BLAST-searched against alfalfa unigenes reported by O’Rourke *et al.* (2015) using the BLASTN program and an E-value set at 1e-20. Meanwhile, these unigenes were also BLAST-searched against combined databases of Arabidopsis, rice, soybean, and *M. truncatula* protein sequences using BLASTX program for functional annotation (the E-value was set at 1e-5). Alfalfa unigenes were assigned with Gene Ontology (GO) annotations based on their corresponding homologs in the combined database, and GO enrichment analysis was displayed using WEGO website (Ye *et al.*, 2006). In addition, alfalfa unigenes were scanned using the iTAK pipeline to identify transcription factors (Jin *et al.*, 2014).

### Expression quantification and differential expression analysis

RNA-seq reads were mapped to alfalfa unigenes using TopHat software (Trapnell *et al.*, 2009), and unigene expressions were estimated as FPKM values (fragments per kilobase of exon per million fragments mapped) using Cufflinks software (Trapnell *et al.*, 2012). EdgeR (Robinson *et al.*, 2010) was used for identifying differentially expressed unigenes with fold changes ≥ 2 or ≤ 0.5, with an adjusted *p*-value of ≤ 0.01. For AP2/ERF TFs, unigenes were BLASTN-searched against *M. truncatula* AP2/ERF mRNAs, and their classification and homologous genes were determined and characterized.

### QRT-PCR analysis of AP2/ERF transcript factor expression

To validate RNA-seq results, qRT-PCR analysis was performed as follows: first, total RNA was isolated from alfalfa samples as previously described, and cDNA was synthesized using the PrimeScript RT reagent Kit (Toyobo, Shanghai, China). Reat-time qRT-PCR detection was performed on a LightCycler 96 System (Roche, Rotkreuz, Switzerland) using SYBR Premix Ex TaqTM II (Toyobo, Shanghai, China) according to the manufacturer’s protocol. The PCR program was set 95 °C for 2 min, followed by 40 cycles of 95 °C for 30 s and 55 °C for 30 s, and a final step of 72 °C for 1 min. The experiments were repeated as three biological replicates, each run as three technical replicates. Ten MsERF genes were randomly selected and their primers were designed for qRT-PCR detection (Table S1). Relative expression of the 10 MsERF genes was calculated and determined based on the 2^-^ΔΔCT method using GAPDH as reference gene.

## Results

### Transcriptome assembly and annotation of alfalfa

**Figure 1:**
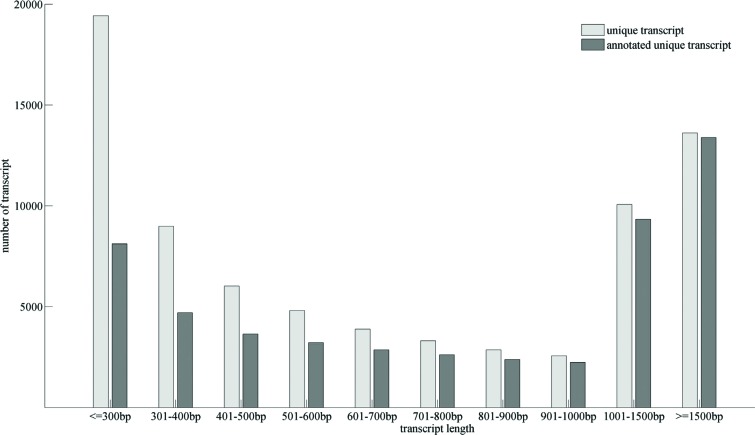
Length distribution of alfalfa unique transcripts.

**Figure 2:**
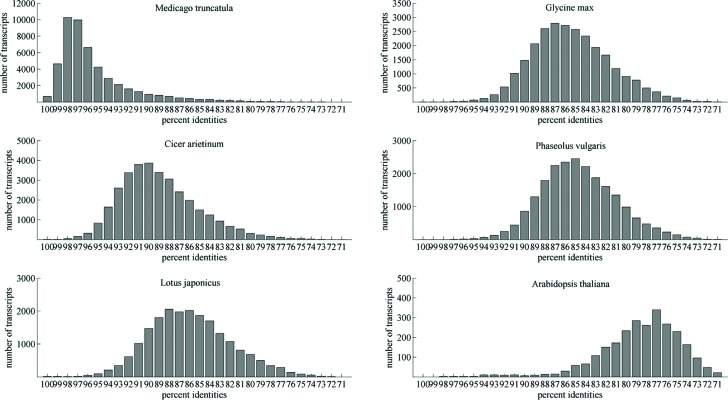
Sequence identity distribution of alfalfa unique transcripts to other plants.

To explore gene profiles of alfalfa response to freezing stress, three transcriptome libraries were designed for RNA-seq. A total of 66 million reads were generated from the three cDNA libraries; all raw and processed data were deposited in the NCBI database (accession number: SRR2529480-82). After cleaning of low quality raw reads and *de novo* assembling, the dataset in total obtained from RNA-seq represented 75,551 alfalfa unigenes. The mean size of alfalfa unigenes is 889 bp, with an N50 value of 1425 bp, as shown in Table 1. By BLASTN searches against previously deposited alfalfa unigenes (O’Rourke *et al.*, 2015), we found that our RNA-seq results had higher similarity with alfalfa unigenes than with cDNA sequences from *M. truncatula*, (Figure S1), implying that the RNA-seq results were highly credible. In addition, the BLASTN results also confirmed that the alfalfa unigenes were highly repeatable. To annotate alfalfa unigenes, we BLASTX-searched against combined databases, and 52,502 (69.5%) unigenes were identified with significant hits. The percentage of unigenes with annotation was positively correlated with the length of the unigenes (Figure 1). In addition, we performed BLASTN searches against mRNAs from Arabidopsis, *Medicago truncatula*, and other legumes for estimating the genetic similarity with other plants. The results showed that alfalfa unigenes had the highest genetic similarity with *M. truncatula* (65.1%, 49,216/75,551), followed by *Cicer arietinum* (44.2%, 33,356/75,551), *Glycine max* (35.0%, 26,464/75,551), *Phaseolus vulgaris* (29.1%, 21,983/75,551), *Lotus japonicus* (27.3%, 20,651/75,551), while Arabidopsis was the lowest (3.5%, 2,631/75,551); see Figure 2.

**Table 1:** Summary of *de novo* assembled alfalfa transcriptome.

Data type	Number
Total sequence	75,551
Number of sequences in 201-500 bp	34,430
Number of sequences in 500-1000 bp	17,435
Number of sequences more than 1000 bp	23,686
Minimal length (bp)	201
Maximal length (bp)	12,056
N50 (bp)	1,425
Average length (bp)	889

### Identification and function annotation analysis of cold and freezing responsive transcripts

**Figure 3:**
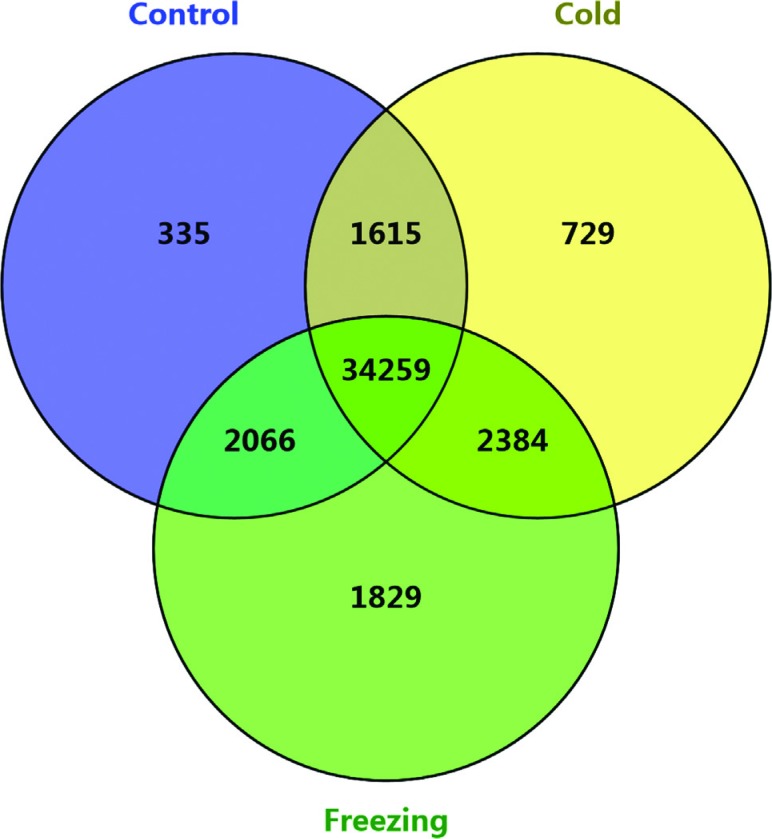
Diagrammatic distribution of alfalfa expressed transcripts in different conditions.

**Figure 4:**
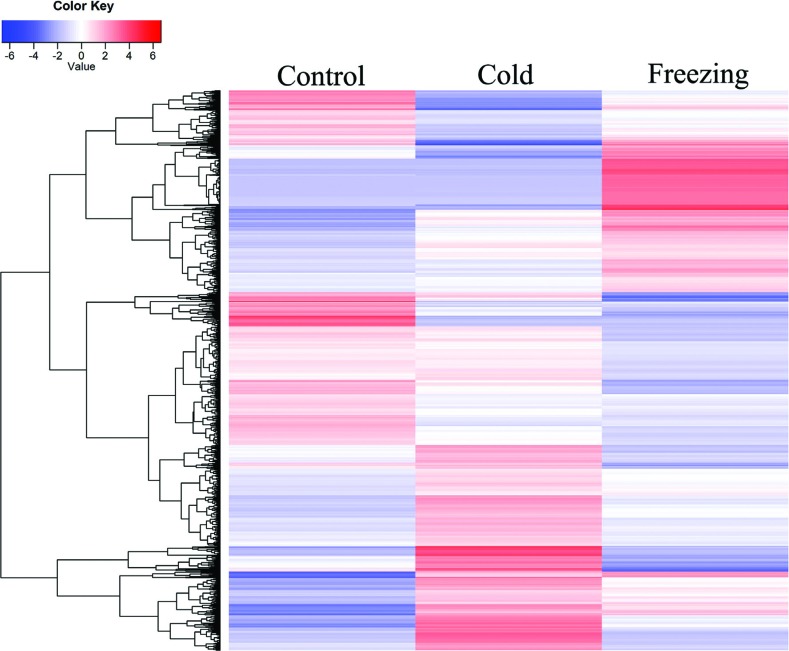
Heatmap showing expression profiles of differentially expressed transcripts in response to cold and/or freezing stress.

To obtain a global view of freezing-responsive gene expression in alfalfa, we aligned reads to transcripts using TopHat2 software. There were 38,275, 38,987, and 40,538 unigenes expressed (FPKM value > 1) in control, cold and freezing stress groups, respectively, most of them were commonly expressed in the three conditions (Figure 3). Using Cufflinks software we identified 3,260 and 3,593 differentially expressed transcripts in response to cold and freezing stress, respectively. To determine their expression profiles in detail, 5,767 transcripts differentially expressed were clustered (Figure 4). The results showed that most of them were only influenced by either cold or freezing stress, and few were influence by both cold and freezing stresses (Figure S2), implying different regulation mechanism responses to cold and freezing stresses in alfalfa.

**Figure 5:**
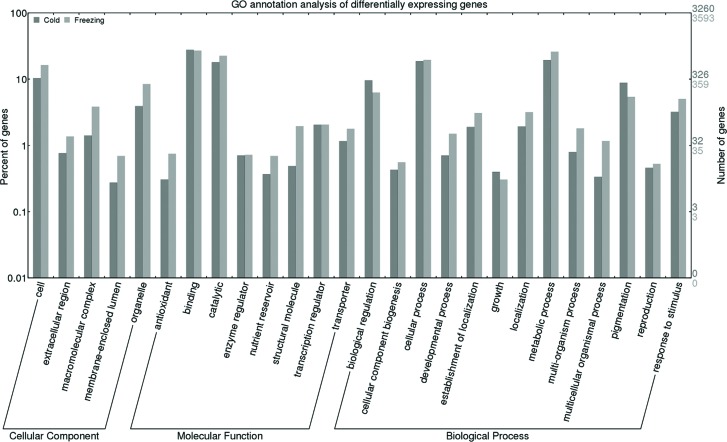
GO annotation results of alfalfa unique transcripts.

By BLAST search analysis, GO terms were assigned to these differentially expressed transcripts, and enrichment analysis for each GO term was performed using the WEGO and topGO package (Figure 5 and Table S2). In the biological process category, we identified highly enriched GO terms involved in stress regulation, including GO:0050896 (response to stimulus), GO:0006950 (response to stress), GO:0009409 (response to cold), which have been well investigated in response to abiotic stress in other plants. Similarly, the most enriched molecular functions were binding (GO:0005488), catalytic activity (GO:0003824), antioxidant activity (GO:0016209), transcription regulator activity (GO:0030528), and transporter activity (GO:0005215), indicating that the transcription regulation, antioxidant and transport systems play important roles in protecting alfalfa from cold and/or freezing stress. Importantly, nodule morphogenesis (GO:0009878) was identified as highly enriched in freezing-response process, but not in cold-response process, implying that freezing tolerance was likely determined by nodulation process in alfalfa.

### Transcription factors involved in cold and/or freezing stress

**Figure 6:**
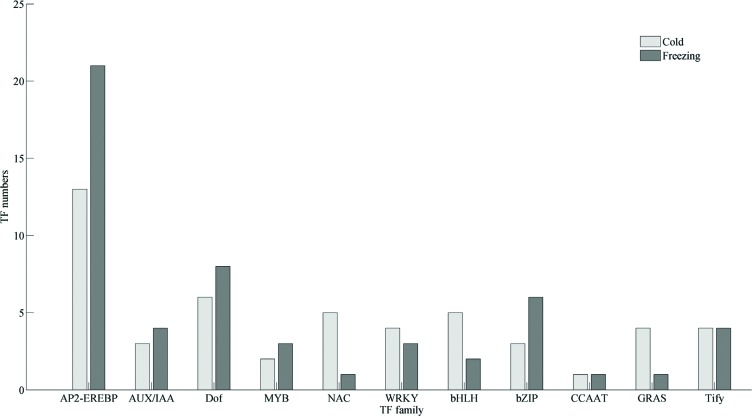
Distribution of transcription factors differentially expressed in response to cold and/or freezing stress by gene family.

**Figure 7:**
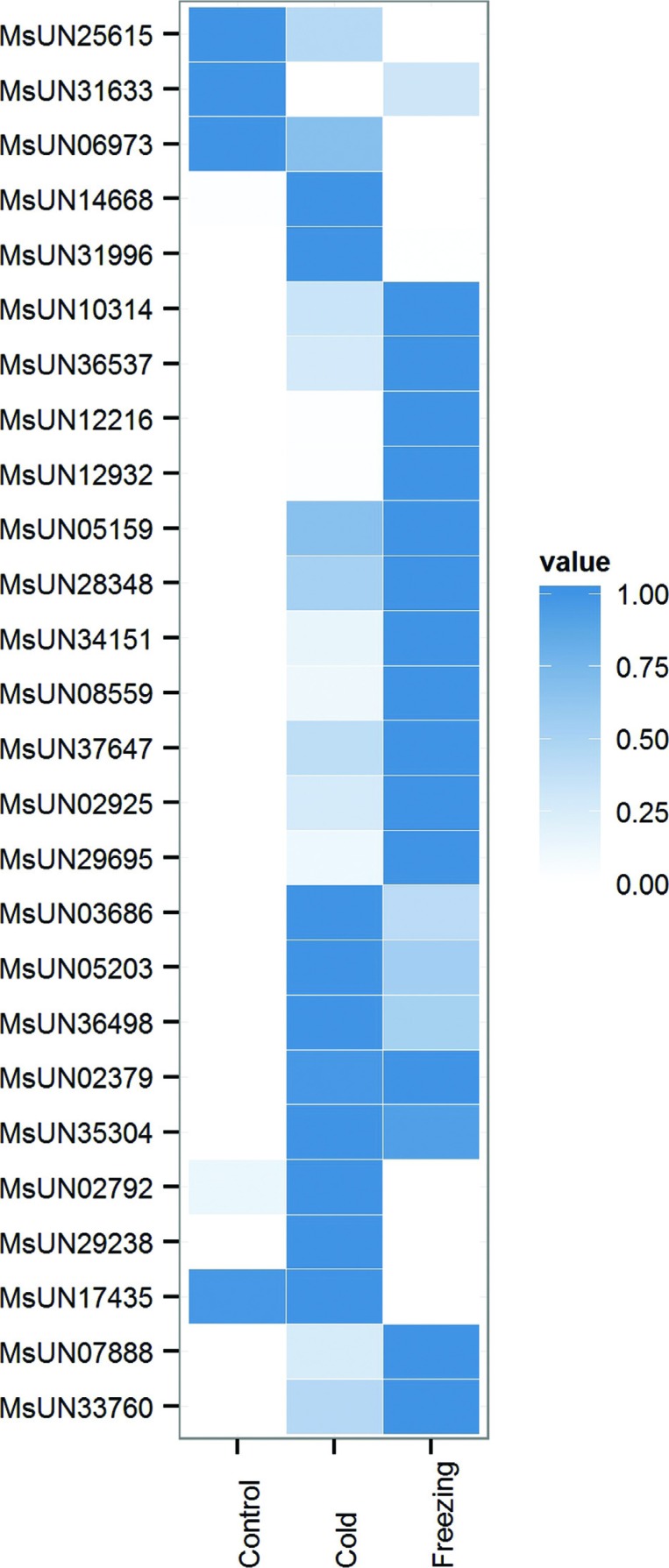
Expression profile of AP2/ERF transcript factors differentially expressed in response to cold and/or freezing stress.

To determine the transcriptional regulation process in detail, the iTAK pipeline was employed for characterizing transcription factors (TF) from alfalfa unigenes. In total, we have characterized 2,138 TFs classified into 79 different families. According to their expression, 158 TFs were identified to be responsive to cold and/or freezing stress (Figure 6), many of which have been previously determined with important roles in the response to abiotic stress, such as AP2-EREBP (also named as AP2/ERF TFs), CCAAT, WRKY, MYB, bZIP, bHLH, NAC, and AUX/IAA. Among these TFs, 26 members were identified as AP2-EREBP TFs (Figure 7), which were responsive to cold and/or freezing stress, and were BLASTN-searched against *M. truncatula* AP2/ERF genes for investigating their function in detail. Twenty unigenes were homologous to MtERF genes, and most of them (17) were classified into the DREB subfamily, which were also named as CBF genes (Table S3). According to homology search results, we identified nine CBF unigenes matching the CBF cluster on chromosome six, named Mt-FTQTL6 site, which is known to confer freezing tolerance trait in *M. truncatula*. Their high transcript levels potentially suggested that there is a similar CBF cluster contributing to freezing tolerance in alfalfa.

**Figure 8:**
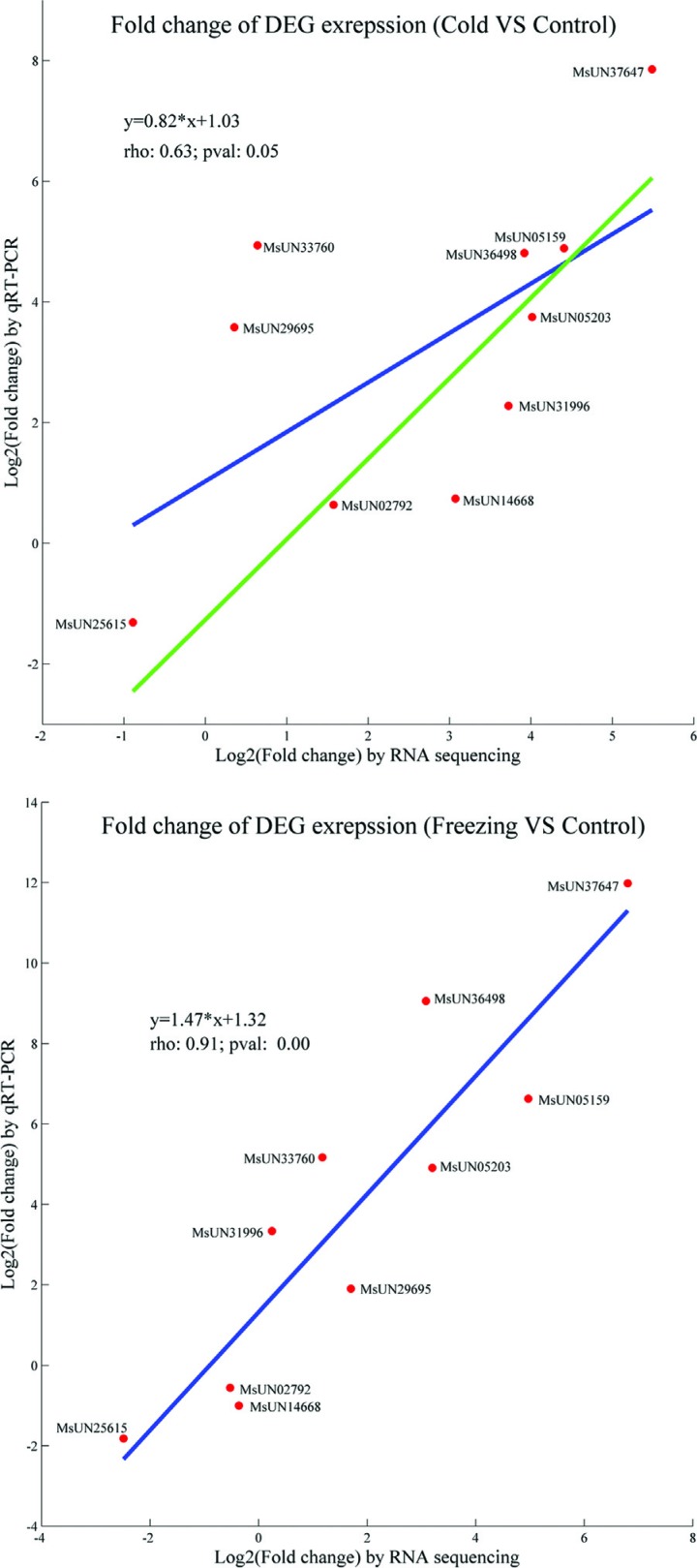
Comparison of the expression of ten MsERF genes between RNA-seq and qRT-PCR platforms in response to cold and freezing stress. Red dots are plot-based fold changes of each MsERF gene between the abundance from transcriptome sequencing and qRT-PCR detection. The line correction relationship was computed based on the expression of ten MsERF genes (blue line), while the green line was computed based on eight MsERF genes, eliminating MsUN29695 and MsUN33760.

To validate the RNA-seq results, we performed qRT-PCR analysis for ten MsERF TF genes in response to cold and freezing stress. The results showed that their expression profiles were highly consistent between the RNA-seq platform and the qRT-PCR method (Figure 8 and Figure S3). In freezing stress, the correlation coefficient of the qRT-PCR validations and the RNA-seq results was as high as 0.91 (*p* < 0.01), while the coefficient was 0.63 in cold stress condition. The deviation was mostly attributed to two unigenes, MsUN29695 and MsUN33760, which elimination would increase the correlation to 0.90. In addition, the PCR products were evaluated by agarose gel electrophoresis, confirming our results. Overall, the qRT-PCR validation confirmed that our RNA-seq results were highly reliable.

## Discussion

Winter weather is a major limitation for the utilization of alfalfa, especially in northern climates. However, freezing tolerance of alfalfa is a complex trait determined by many genetic components and the environment (Castonguay *et al.*, 2013). To investigate the complex genetic regulation mechanisms of freezing tolerance in alfalfa, we performed RNA-seq analysis of alfalfa under cold and freezing stress. We identified several genes involved in the metabolism and regulation process of freezing tolerance, which are discussed in detail below.

### Phytohormone signals associated with freezing response

In perennial plants, the phytohormones ABA, auxin, and GA are known to play important roles in adaption to cold stress by regulating plant growth processes. For example, *Populus* trees were characterized as having lower levels of ABA, auxin, and GA in the dormancy process, which controls plant growth and development in response to winter strain (Cooke *et al.*, 2012). In our study, we did not identify the levels of these phytohormones, but we found transcriptional levels of functional genes known to respond to them (Table S4). Most of the functional genes were repressed by cold and/or freezing stresses, implying their roles in controlling alfalfa growth under low temperature stresses (Zabotin *et al.*, 2009). This finding was consistent with previous reports in perennial trees, which indicated that alfalfa may adopt similar activity-dormancy cycles in adapting to winter hardness. Besides these dormancy-related phytohormones, we also identified abiotic stress-responsive phytohormones, such as BA and JA, which were characterized as positive regulatory genes in response to abiotic stresses, such as cold and/or freezing stresses. Interestingly, all transcripts that respond to JA hormones, were up-regulated by cold stress, and repressed or normally expressed under freezing stress. These transcripts encode JAZ proteins, which were identified as physically interactive with ICE1 and ICE2 transcription factors, repressing ICE-CBF/DREB1 cascade in plants (Robson *et al.*, 2010; Hu *et al.*, 2013). In alfalfa, the expression of JAZ proteins diverged between cold and freezing tests, while in *M. truncatula*, JAZ proteins were highly expressed under both cold and freezing stresses (Figure S4). The results suggest that alfalfa adopts other regulation pathways to reduce expression of JAZ proteins, release function of ICE1, and improve freezing tolerance. However, the exact roles of JAZ in response to freezing stress remain to be elucidated, and their function should be characterized in future.

### Transcription factors involved in freezing response

Plant TFs play important roles in response to abiotic stresses, including cold and freezing. In the present study, we identified 158 TFs differentially expressed in alfalfa under cold and/or freezing stress. Among these TFs, the AP2/ERF family contains most members, implying their critical roles in alfalfa response to cold and/or freezing conditions. Other common TF families (Figure 6) involved in plant abiotic stress process, such as WRKY, NAC, bZIP, bHLH, AUX/IAA, etc., were also identified in the alfalfa transcriptomes, which is consist with previous reports in other plants. CCAAT and GRAS TFs that participate in the nodulation process of alfalfa were also differentially expressed under low temperatures. In our previous study we characterized miRNA genes, such as miRNA169, that regulate the nodulation process by targeting CCAAT TFs under cold and/or freezing stress (Shu *et al.*, 2016). These results indicated that the nodulation process may critically contribute to freezing tolerance traits of alfalfa, which have also been investigated in other Medicago species.

### CBFs regulation function in freezing response

In plants, CBF genes, also known as DREB genes, belong to the AP2/ERF TF superfamily, which binds to the DRE/CRT regulatory element, regulates expression of downstream functional genes, such as RD29, COR47, KIN7, and others (Takumi *et al.*, 2008; Thomashow, 2010; Kim *et al.*, 2015). CBF and related genes constitute CBF pathways, which confer important roles in plant cold accumulation process and freezing tolerance trait. In *M. truncatula*, Tayeh *et al.* (2013) detected a major QTL on chromosome six (Mt-FTQTL6) conferring freezing tolerance, and we have identified twelve CBF genes closely associated with this site. Previous RNA-seq results have confirmed that six of them positively respond to cold and/or freezing stress in *M. truncatula* (Shu *et al.*, 2015). In alfalfa, Castonguay *et al.* (2010) have reported a sequence-related amplified polymorphism marker associated with freezing tolerance, which is matched to flanking sequences of the Mt-FTQTL6 site. These findings suggest that there are freezing tolerance controlling sites, with clustered CBF genes, which are highly syntenic between the *M. truncatula* and *M. sativa* genomes. However, these CBF genes have not been characterized in *M. sativa*, especially their regulatory roles in freezing tolerance determination. In the present study, we have identified 15 transcripts highly similar to CBF genes associated withthe Mt-FTQTL6 site, nine of them being positively regulated by cold and/or freezing stress (Table S3). These results confirm a CBF cluster similar to the one present in *M. sativa* and indicate its potential role in freezing tolerance of alfalfa. Because of the lack of a genome sequence for *M. sativa*, the molecular mechanism of the CBF cluster response to freezing stress in alfalfa remains to be determined in future.

## Conclusions

In the present study a transcriptome sequencing analysis of alfalfa response to cold and freezing stresses was performed that identified 75,551 transcripts.. A number of cold- and/or freezing-responsive transcripts were characterized, mainly involved in response to stress, signal transduction, transcriptional regulation, hormone signaling pathways, etc.. Especially nine CBF genes were characterized as having a positive response to cold and/or freezing stresses, which indicates that the CBF cluster conferring freezing tolerance is highly conserved between *M. truncatula* and *M. sativa*. These results should be helpful in the exploration of an adaption mechanism of alfalfa to freezing stress, and could be introduced into cultivated alfalfa for improvement of freezing tolerance in future.

## Supplementary material

The following online material is available for this article:

Figure S1 - Sequence identity distribution of alfalfa unigenes.

Figure S2 - Diagrammatic distribution of alfalfa transcripts.

Figure S3 - qRT-PCR validation of 10 MsERF genes.

Figure S4 - Expression models of four JAZ genes.

Table S1 - List of qRT-PCR validation primers used in the present study.

Table S2 - Functional enrichment analysis of GO terms.

Table S3 - Transcripts homologous to AP2/ERF genes differentially expressed.

Table S4 - Hormone-related transcripts that were differentially expressed.
