# Supplementary material for: Transcriptome sequencing analysis of alfalfa reveals CBF genes potentially playing important roles in response to freezing stress
Source: Genet Mol Biol. 2017 Nov 6;40(4):824–33. doi: 10.1590/1678-4685-GMB-2017-0053 (PMC5738619; doi:10.1590/1678-4685-GMB-2017-0053)
Supplement: Supplementary file 1 [file 1415-4757-gmb-1678-4685-GMB-2017-0053-Suppl05.pdf]

Supplementary Material to “Transcriptome sequencing analysis of alfalfa reveals CBF genes potentially playing important roles in response to freezing stress”

Table S1 - List of qRT-PCR validation primers used in the present study.

Gene	Forward primer	Reverses primer
MsUN02792	TAGTACAGCAAAGCGAATCA	ATTTCTGCTACCCACTTACC
MsUN37647	GTTCCATAGTGATACCTCATCA	ACGACATTGACACTGTTGTA
MsUN33760	CTTCTCCTCACTCCTCTGTA	TCCTTGGTTTTGAAGCTGAT
MsUN31996	CTTCGGTGTCAAAGAGAGAA	GTTGAAGTGTTTGGCTTACT
MsUN25615	ATGCATGGAGTTGCTACATT	AACACACTATTGATGGTGGT
MsUN29695	TGAGATTTGGGGGAGATTTG	AGGATGAAGGGAAAAAGTGG
MsUN05203	TATAGGGGTGTGAGGAAGAG	GAATAGGAAGCCTCCACAC
MsUN36498	GTTCCATAGTGATACCTCATCA	ACGACATTGACACTGTTGTA
MsUN14668	GCGTGTTTAGGGTTTACAAA	CGTGGAGAAGTTTTTGGAAG
MsUN05159	CCACTAAAAATGGTCCAGTAC	ATAGCTGGGGACATGAGTAT
GAPDH	TAAGGGTGGTGCCAAGAAGGT	AGCAAGAGGAGCAAGGCAGTT
