# Supplementary material for: Transcriptome sequencing analysis of alfalfa reveals CBF genes potentially playing important roles in response to freezing stress
Source: Genet Mol Biol. 2017 Nov 6;40(4):824–33. doi: 10.1590/1678-4685-GMB-2017-0053 (PMC5738619; doi:10.1590/1678-4685-GMB-2017-0053)

Supplementary Material to "Transcriptome sequencing analysis of alfalfa reveals CBF genes potentially playing important roles in response to freezing stress"

Figure S1 - Sequence identity distribution of alfalfa unigenes to *M. truncatula* cDNA and previous alfalfa unigenes.

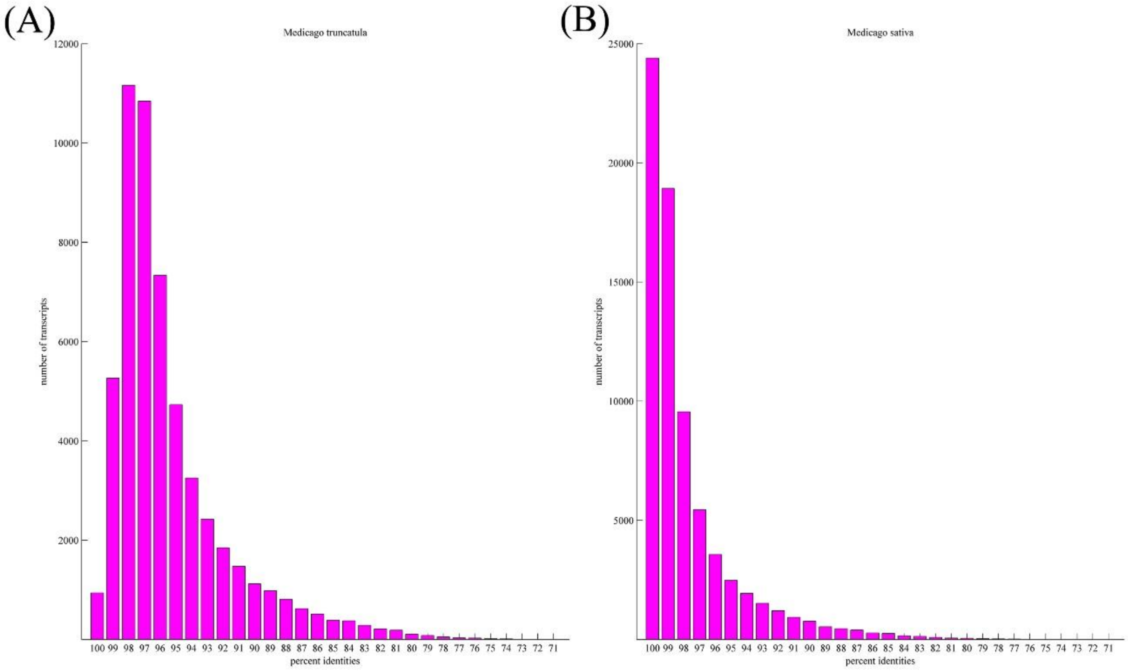

Supplement: Supplementary file 2 [file 1415-4757-gmb-1678-4685-GMB-2017-0053-Suppl01.pdf]
