# Supplementary material for: Transcriptome sequencing analysis of alfalfa reveals CBF genes potentially playing important roles in response to freezing stress
Source: Genet Mol Biol. 2017 Nov 6;40(4):824–33. doi: 10.1590/1678-4685-GMB-2017-0053 (PMC5738619; doi:10.1590/1678-4685-GMB-2017-0053)
Supplement: Supplementary file 4 [file 1415-4757-gmb-1678-4685-GMB-2017-0053-Suppl06.pdf]

Supplementary Material to “Transcriptome sequencing analysis of alfalfa reveals CBF genes potentially playing important roles in response to freezing stress”

Table S2 - Functional enrichment analysis of GO terms using topGO in alfalfa response to cold and/or freezing stress.

Group	GO ID	Term Annotated	Total Transcripts	Significant Transcripts	P-value
Cold BP	GO:0023052	signaling	1337	187	8.00E-30
	GO:0044700	single organism signaling	1337	187	8.00E-30
	GO:0007165	signal transduction	1332	186	1.40E-29
	GO:0050794	regulation of cellular process	2452	277	1.20E-28
	GO:0007154	cell communication	1501	195	6.10E-27
	GO:0050789	regulation of biological process	2679	288	2.90E-26
	GO:0065007	biological regulation	2798	293	6.90E-25
	GO:0050896	response to stimulus	2738	286	4.80E-24
	GO:0051716	cellular response to stimulus	1588	191	2.60E-22
	GO:0000272	polysaccharide catabolic process	15	9	3.10E-08
	GO:0044699	single-organism process	6718	481	5.30E-08
	GO:0033993	response to lipid	111	23	1.10E-07
	GO:0005976	polysaccharide metabolic process	169	29	2.10E-07
	GO:0001101	response to acid	182	29	1.00E-06
	GO:1901700	response to oxygen-containing compound	269	37	1.50E-06
	GO:0044763	single-organism cellular process	4436	328	1.80E-06
	GO:0044262	cellular carbohydrate metabolic process	232	32	7.10E-06

Group	GO ID	Term Annotated	Total Transcripts	Significant Transcripts	P-value
	GO:0009739	response to gibberellin	26	9	1.10E-05
	GO:0097305	response to alcohol	91	16	8.00E-05
	GO:0006414	translational elongation	85	15	0.00013
	GO:0005975	carbohydrate metabolic process	824	75	0.00013
	GO:0009741	response to brassinosteroid	15	6	0.00013
	GO:0042545	cell wall modification	78	14	0.00017
	GO:0006073	cellular glucan metabolic process	142	20	0.00027
	GO:0044042	glucan metabolic process	142	20	0.00027
	GO:0051252	regulation of RNA metabolic process	932	81	0.00032
	GO:0009828	plant-type cell wall loosening	31	8	0.00034
	GO:0009827	plant-type cell wall modification	39	9	0.00036
	GO:0071555	cell wall organization	95	15	0.00045
	GO:0006355	regulation of transcription, DNA-templat...	930	80	0.00047
	GO:2001141	regulation of RNA biosynthetic process	930	80	0.00047
	GO:0045229	external encapsulating structure organiz...	97	15	0.00056
	GO:0009725	response to hormone	309	33	0.00077
	GO:0010556	regulation of macromolecule biosynthetic...	949	80	0.00085
	GO:2000112	regulation of cellular macromolecule bio...	949	80	0.00085
	GO:0031326	regulation of cellular biosynthetic proc...	952	80	0.00093
	GO:0009889	regulation of biosynthetic process	953	80	0.00096
	GO:0044264	cellular polysaccharide metabolic proces...	158	20	0.00107
	GO:0000902	cell morphogenesis	93	14	0.00111
	GO:0005984	disaccharide metabolic process	54	10	0.00112
	GO:0009719	response to endogenous stimulus	319	33	0.00132
	GO:0032989	cellular component morphogenesis	95	14	0.00137
	GO:0009826	unidimensional cell growth	65	11	0.0014
	GO:0019219	regulation of nucleobase-containing comp...	981	81	0.00144
	GO:0009664	plant-type cell wall organization	56	10	0.0015

Group	GO ID	Term Annotated	Total Transcripts	Significant Transcripts	P-value
	GO:0051171	regulation of nitrogen compound metaboli...	983	81	0.00152
	GO:0009651	response to salt stress	118	16	0.00159
	GO:0051607	defense response to virus	10	4	0.00192
	GO:0009415	response to water	68	11	0.00204
	GO:0055114	oxidation-reduction process	1469	113	0.00221
	GO:0009987	cellular process	9318	597	0.00248
	GO:0009737	response to abscisic acid	80	12	0.00252
	GO:0010150	leaf senescence	11	4	0.00288
	GO:0010260	organ senescence	11	4	0.00288
	GO:0006970	response to osmotic stress	125	16	0.0029
	GO:0031323	regulation of cellular metabolic process	1009	81	0.00309
	GO:0080090	regulation of primary metabolic process	1009	81	0.00309
	GO:0060560	developmental growth involved in morphog...	72	11	0.00324
	GO:0042221	response to chemical	625	54	0.00342
	GO:0009086	methionine biosynthetic process	6	3	0.00362
	GO:0010468	regulation of gene expression	1002	80	0.00377
	GO:0002252	immune effector process	12	4	0.00412
	GO:0009311	oligosaccharide metabolic process	64	10	0.00415
	GO:0071669	plant-type cell wall organization or bio...	64	10	0.00415
	GO:0019222	regulation of metabolic process	1172	91	0.00453
	GO:0060255	regulation of macromolecule metabolic pr...	1025	81	0.00464
	GO:0006351	transcription, DNA-templated	1055	83	0.0047
	GO:0010033	response to organic substance	413	38	0.00474
	GO:0016052	carbohydrate catabolic process	87	12	0.00509
	GO:0009414	response to water deprivation	66	10	0.0052
	GO:0009067	aspartate family amino acid biosynthetic...	20	5	0.0053
	GO:0032774	RNA biosynthetic process	1064	83	0.00584

Group	GO ID	Term Annotated	Total Transcripts	Significant Transcripts	P-value
	GO:0016049	cell growth	100	13	0.00604
	GO:0006555	methionine metabolic process	7	3	0.00605
	GO:0010035	response to inorganic substance	221	23	0.00615
	GO:0005985	sucrose metabolic process	29	6	0.00623
	GO:0030244	cellulose biosynthetic process	68	10	0.00645
	GO:0009066	aspartate family amino acid metabolic pr...	21	5	0.00662
	GO:0034637	cellular carbohydrate biosynthetic proce...	149	17	0.00712
	GO:0030243	cellulose metabolic process	69	10	0.00715
	GO:0048589	developmental growth	80	11	0.00732
	GO:0009269	response to desiccation	15	4	0.00986
Cold MF	GO:0043531	ADP binding	1640	235	< 1e-30
	GO:0016702	oxidoreductase activity, acting on singl...	71	25	1.20E-13
	GO:0016701	oxidoreductase activity, acting on singl...	74	25	3.40E-13
	GO:0016161	beta-amylase activity	9	9	8.70E-12
	GO:0030554	adenyl nucleotide binding	4287	350	2.30E-11
	GO:0005515	protein binding	5903	456	2.60E-11
	GO:0032559	adenyl ribonucleotide binding	4248	346	4.30E-11
	GO:0032553	ribonucleotide binding	4606	368	9.60E-11
	GO:0016160	amylase activity	13	10	1.20E-10
	GO:0097367	carbohydrate derivative binding	4627	368	1.70E-10
	GO:0017076	purine nucleotide binding	4621	366	3.30E-10
	GO:0001883	purine nucleoside binding	4579	362	5.50E-10
	GO:0032549	ribonucleoside binding	4579	362	5.50E-10
	GO:0032550	purine ribonucleoside binding	4579	362	5.50E-10
	GO:0032555	purine ribonucleotide binding	4579	362	5.50E-10
	GO:0001882	nucleoside binding	4582	362	6.00E-10
	GO:0000166	nucleotide binding	4941	381	3.60E-09
	GO:1901265	nucleoside phosphate binding	4941	381	3.60E-09

Group	GO ID	Term Annotated	Total Transcripts	Significant Transcripts	P-value
	GO:0036094	small molecule binding	5012	382	1.40E-08
	GO:0043167	ion binding	7091	514	1.60E-08
	GO:0043168	anion binding	4960	374	7.10E-08
	GO:0016758	transferase activity, transferring hexos...	535	57	1.30E-05
	GO:0004857	enzyme inhibitor activity	123	20	3.50E-05
	GO:0003746	translation elongation factor activity	68	14	3.70E-05
	GO:1901363	heterocyclic compound binding	7655	518	8.80E-05
	GO:0097159	organic cyclic compound binding	7660	518	9.40E-05
	GO:0016762	xyloglucan:xyloglucosyl transferase acti...	34	9	0.00012
	GO:0005509	calcium ion binding	236	29	0.00016
	GO:0016757	transferase activity, transferring glyco...	642	61	0.00018
	GO:0005310	dicarboxylic acid transmembrane transpor...	3	3	0.00021
	GO:0051213	dioxygenase activity	269	31	0.0003
	GO:0045735	nutrient reservoir activity	65	12	0.00038
	GO:0004553	hydrolase activity, hydrolyzing O-glycos...	452	45	0.00046
	GO:0046906	tetrapyrrole binding	463	45	0.00077
	GO:0003871	5-methyltetrahydropteroyltriglutamate-ho...	5	3	0.00189
	GO:0042085	5-methyltetrahydropteroyltri-L-glutamate...	5	3	0.00189
	GO:0016798	hydrolase activity, acting on glycosyl b...	486	45	0.00203
	GO:0016746	transferase activity, transferring acyl ...	302	31	0.00206
	GO:0005488	binding	13784	867	0.00236
	GO:0030599	pectinesterase activity	60	10	0.00256
	GO:0016491	oxidoreductase activity	1657	125	0.00281
	GO:0008135	translation factor activity, nucleic aci...	115	15	0.00322
	GO:0010181	FMN binding	27	6	0.00429
	GO:0020037	heme binding	438	40	0.0044
	GO:0001071	nucleic acid binding transcription facto...	767	62	0.00796
	GO:0003700	sequence-specific DNA binding transcript...	767	62	0.00796

Group	GO ID	Term Annotated	Total Transcripts	Significant Transcripts	P-value
	GO:0004866	endopeptidase inhibitor activity	60	9	0.00836
	GO:0030414	peptidase inhibitor activity	60	9	0.00836
	GO:0061134	peptidase regulator activity	60	9	0.00836
	GO:0061135	endopeptidase regulator activity	60	9	0.00836
Cold CC	GO:0005618	cell wall	249	37	8.60E-12
	GO:0030312	external encapsulating structure	249	37	8.60E-12
	GO:0005576	extracellular region	156	25	4.70E-09
	GO:0009505	plant-type cell wall	53	13	1.60E-07
	GO:0048046	apoplast	97	15	9.40E-06
	GO:0071944	cell periphery	921	58	7.00E-04
Freezing BP	GO:0015979	photosynthesis	103	39	2.90E-17
	GO:0009877	nodulation	37	22	2.00E-15
	GO:0009878	nodule morphogenesis	37	22	2.00E-15
	GO:0044111	development involved in symbiotic intera...	37	22	2.00E-15
	GO:0008152	metabolic process	9998	934	3.30E-13
	GO:0009886	post-embryonic morphogenesis	61	25	1.80E-12
	GO:0006950	response to stress	985	137	9.20E-11
	GO:0055114	oxidation-reduction process	1469	185	2.10E-10
	GO:0019684	photosynthesis, light reaction	39	18	2.10E-10
	GO:0009405	pathogenesis	29	15	9.20E-10
	GO:0044403	symbiosis, encompassing mutualism throug...	80	25	1.60E-09
	GO:0044419	interspecies interaction between organis...	80	25	1.60E-09
	GO:0009628	response to abiotic stimulus	520	78	5.30E-08
	GO:0009653	anatomical structure morphogenesis	204	39	3.00E-07
	GO:0051704	multi-organism process	466	69	5.10E-07
	GO:0044710	single-organism metabolic process	3113	320	5.10E-07
	GO:0005976	polysaccharide metabolic process	169	33	1.50E-06

Group	GO ID	Term Annotated	Total Transcripts	Significant Transcripts	P-value
	GO:0009765	photosynthesis, light harvesting	26	11	2.20E-06
	GO:0009767	photosynthetic electron transport chain	11	7	5.30E-06
	GO:0006869	lipid transport	50	15	5.30E-06
	GO:0010876	lipid localization	50	15	5.30E-06
	GO:0006952	defense response	196	35	6.00E-06
	GO:0009266	response to temperature stimulus	206	36	7.30E-06
	GO:0006073	cellular glucan metabolic process	142	28	7.30E-06
	GO:0044042	glucan metabolic process	142	28	7.30E-06
	GO:0006979	response to oxidative stress	198	35	7.50E-06
	GO:0042545	cell wall modification	78	19	9.40E-06
	GO:0080167	response to karrikin	54	15	1.50E-05
	GO:0010035	response to inorganic substance	221	37	1.50E-05
	GO:0009637	response to blue light	17	8	2.10E-05
	GO:0006412	translation	686	85	3.80E-05
	GO:0006091	generation of precursor metabolites and ...	141	26	5.10E-05
	GO:0044264	cellular polysaccharide metabolic proces...	158	28	5.70E-05
	GO:0009773	photosynthetic electron transport in pho...	7	5	6.10E-05
	GO:0000272	polysaccharide catabolic process	15	7	7.70E-05
	GO:0050896	response to stimulus	2738	271	8.30E-05
	GO:0009058	biosynthetic process	2947	289	8.90E-05
	GO:0071555	cell wall organization	95	19	0.00017
	GO:0045229	external encapsulating structure organiz...	97	19	0.00023
	GO:1901700	response to oxygen-containing compound	269	39	0.00023
	GO:0010218	response to far red light	23	8	0.00028
	GO:0033993	response to lipid	111	20	0.0005
	GO:0009828	plant-type cell wall loosening	31	9	0.00054
	GO:0018198	peptidyl-cysteine modification	6	4	0.00054
	GO:0009607	response to biotic stimulus	232	33	0.00093

Group	GO ID	Term Annotated	Total Transcripts	Significant Transcripts	P-value
	GO:0044262	cellular carbohydrate metabolic process	232	33	0.00093
	GO:0009409	response to cold	102	18	0.00119
	GO:0005975	carbohydrate metabolic process	824	90	0.00165
	GO:0042221	response to chemical	625	71	0.00184
	GO:0009827	plant-type cell wall modification	39	9	0.00316
	GO:0010114	response to red light	26	7	0.00358
	GO:1901576	organic substance biosynthetic process	2736	256	0.00364
	GO:0016052	carbohydrate catabolic process	87	15	0.00372
	GO:0009408	response to heat	122	19	0.00391
	GO:0044711	single-organism biosynthetic process	666	73	0.00398
	GO:0015977	carbon fixation	21	6	0.00503
	GO:0009741	response to brassinosteroid	15	5	0.00503
	GO:0097305	response to alcohol	91	15	0.00573
	GO:0010249	auxin conjugate metabolic process	2	2	0.00645
	GO:0006633	fatty acid biosynthetic process	76	13	0.00713
	GO:0016053	organic acid biosynthetic process	244	31	0.00736
	GO:0046394	carboxylic acid biosynthetic process	244	31	0.00736
	GO:0006508	proteolysis	651	70	0.00737
	GO:0006414	translational elongation	85	14	0.00751
	GO:0044249	cellular biosynthetic process	2703	249	0.00879
	GO:0009831	plant-type cell wall modification involv...	17	5	0.00906
	GO:0042547	cell wall modification involved in multi...	17	5	0.00906
	GO:0016485	protein processing	659	70	0.00966
	GO:0051604	protein maturation	659	70	0.00966
Freezing MF	GO:0045735	nutrient reservoir activity	65	25	1.40E-12
	GO:0046906	tetrapyrrole binding	463	77	1.10E-11
	GO:0016491	oxidoreductase activity	1657	192	9.70E-11
	GO:0016168	chlorophyll binding	25	14	2.60E-10

Group	GO ID	Term Annotated	Total Transcripts	Significant Transcripts	P-value
	GO:0016762	xyloglucan:xyloglucosyl transferase acti...	34	16	4.20E-10
	GO:0016701	oxidoreductase activity, acting on singl...	74	23	1.50E-09
	GO:0016702	oxidoreductase activity, acting on singl...	71	22	3.70E-09
	GO:0016746	transferase activity, transferring acyl ...	302	52	7.30E-09
	GO:0003824	catalytic activity	9532	810	2.20E-08
	GO:0020037	heme binding	438	63	2.30E-07
	GO:0016209	antioxidant activity	128	27	5.20E-07
	GO:0004857	enzyme inhibitor activity	123	25	2.80E-06
	GO:0016829	lyase activity	260	39	1.70E-05
	GO:0004601	peroxidase activity	113	22	2.20E-05
	GO:0016684	oxidoreductase activity, acting on perox...	113	22	2.20E-05
	GO:0046872	metal ion binding	2254	216	2.20E-05
	GO:0005506	iron ion binding	361	49	2.40E-05
	GO:0043169	cation binding	2266	216	3.10E-05
	GO:0016747	transferase activity, transferring acyl ...	240	35	7.90E-05
	GO:0030599	pectinesterase activity	60	14	8.90E-05
	GO:0004462	lactoylglutathione lyase activity	12	6	9.80E-05
	GO:0016846	carbon-sulfur lyase activity	17	7	0.00012
	GO:0005198	structural molecule activity	609	70	0.00013
	GO:0016160	amylase activity	13	6	0.00017
	GO:0003735	structural constituent of ribosome	507	60	0.00018
	GO:0016161	beta-amylase activity	9	5	0.00021
	GO:0008289	lipid binding	168	26	0.00024
	GO:0016758	transferase activity, transferring hexos...	535	62	0.00025
	GO:0008135	translation factor activity, nucleic aci...	115	20	0.00026
	GO:0008236	serine-type peptidase activity	227	32	0.00029
	GO:0017171	serine hydrolase activity	227	32	0.00029
	GO:0008233	peptidase activity	623	69	0.00043

Group	GO ID	Term Annotated	Total Transcripts	Significant Transcripts	P-value
	GO:0004175	endopeptidase activity	356	44	0.0005
	GO:0070011	peptidase activity, acting on L-amino ac...	595	66	0.00054
	GO:0004252	serine-type endopeptidase activity	143	22	0.00076
	GO:0008171	O-methyltransferase activity	52	11	0.00119
	GO:0003746	translation elongation factor activity	68	13	0.00121
	GO:0004866	endopeptidase inhibitor activity	60	12	0.00122
	GO:0030414	peptidase inhibitor activity	60	12	0.00122
	GO:0061134	peptidase regulator activity	60	12	0.00122
	GO:0061135	endopeptidase regulator activity	60	12	0.00122
	GO:0051213	dioxygenase activity	269	34	0.0014
	GO:0004553	hydrolase activity, hydrolyzing O-glycos...	452	51	0.00151
	GO:0016830	carbon-carbon lyase activity	80	14	0.00194
	GO:0004222	metalloendopeptidase activity	73	13	0.00236
	GO:0008237	metallopeptidase activity	82	14	0.00246
	GO:0016798	hydrolase activity, acting on glycosyl b...	486	53	0.00259
	GO:0051082	unfolded protein binding	110	17	0.00274
	GO:0016705	oxidoreductase activity, acting on paire...	551	58	0.00369
	GO:0052689	carboxylic ester hydrolase activity	143	20	0.00405
	GO:0016757	transferase activity, transferring glyco...	642	65	0.0054
	GO:0016831	carboxy-lyase activity	55	10	0.00622
	GO:0016984	ribulose-bisphosphate carboxylase activi...	6	3	0.00669
	GO:0016787	hydrolase activity	3115	263	0.00744
	GO:0004124	cysteine synthase activity	12	4	0.00891
	GO:0019904	protein domain specific binding	12	4	0.00891
	GO:0045309	protein phosphorylated amino acid bindin...	12	4	0.00891
	GO:0051219	phosphoprotein binding	12	4	0.00891
Freezing CC	GO:0009579	thylakoid	223	88	< 1e-30
	GO:0044436	thylakoid part	204	82	< 1e-30

Group	GO ID	Term Annotated	Total Transcripts	Significant Transcripts	P-value
	GO:0034357	photosynthetic membrane	191	79	< 1e-30
	GO:0044434	chloroplast part	346	104	< 1e-30
	GO:0044435	plastid part	354	104	< 1e-30
	GO:0042651	thylakoid membrane	160	67	< 1e-30
	GO:0009534	chloroplast thylakoid	145	63	< 1e-30
	GO:0031976	plastid thylakoid	145	63	< 1e-30
	GO:0031984	organelle subcompartment	145	63	< 1e-30
	GO:0009535	chloroplast thylakoid membrane	131	58	< 1e-30
	GO:0055035	plastid thylakoid membrane	131	58	< 1e-30
	GO:0009507	chloroplast	1195	172	6.20E-25
	GO:0009532	plastid stroma	143	51	7.40E-25
	GO:0009570	chloroplast stroma	136	49	3.70E-24
	GO:0009536	plastid	1222	172	8.40E-24
	GO:0005576	extracellular region	156	48	2.60E-20
	GO:0005618	cell wall	249	57	3.50E-17
	GO:0030312	external encapsulating structure	249	57	3.50E-17
	GO:0009526	plastid envelope	174	45	6.70E-16
	GO:0048046	apoplast	97	33	8.60E-16
	GO:0009941	chloroplast envelope	166	42	1.50E-14
	GO:0005623	cell	5785	476	1.90E-14
	GO:0044464	cell part	5785	476	1.90E-14
	GO:0044444	cytoplasmic part	2789	271	6.10E-14
	GO:0009521	photosystem	59	23	7.70E-13
	GO:0005737	cytoplasm	2990	280	2.00E-12
	GO:0044422	organelle part	1119	127	2.70E-10
	GO:0044446	intracellular organelle part	1119	127	2.70E-10
	GO:0009523	photosystem II	37	14	3.90E-08
	GO:0010287	plastoglobule	23	10	7.80E-07

Group	GO ID	Term Annotated	Total Transcripts	Significant Transcripts	P-value
	GO:0031977	thylakoid lumen	29	11	1.10E-06
	GO:0005840	ribosome	531	64	1.60E-06
	GO:0030076	light-harvesting complex	21	9	3.30E-06
	GO:0005622	intracellular	4620	362	4.20E-06
	GO:0044424	intracellular part	4323	342	4.40E-06
	GO:0009654	photosystem II oxygen evolving complex	17	8	5.00E-06
	GO:0009505	plant-type cell wall	53	14	5.70E-06
	GO:0032991	macromolecular complex	1505	139	1.30E-05
	GO:0031967	organelle envelope	375	47	1.50E-05
	GO:0031975	envelope	375	47	1.50E-05
	GO:0009522	photosystem I	21	8	3.30E-05
	GO:0009538	photosystem I reaction center	8	5	5.90E-05
	GO:0010319	stromule	8	5	5.90E-05
	GO:0030529	ribonucleoprotein complex	601	64	8.60E-05
	GO:0009368	endopeptidase Clp complex	9	5	0.00013
	GO:0009840	chloroplastic endopeptidase Clp complex	9	5	0.00013
	GO:0043226	organelle	3773	293	0.00023
	GO:0043229	intracellular organelle	3773	293	0.00023
	GO:0071944	cell periphery	921	85	0.00084
	GO:0019898	extrinsic component of membrane	25	7	0.0009
	GO:0009543	chloroplast thylakoid lumen	20	6	0.00142
	GO:0031978	plastid thylakoid lumen	20	6	0.00142
	GO:0043228	non-membrane-bounded organelle	834	77	0.00147
	GO:0043232	intracellular non-membrane-bounded organ...	834	77	0.00147
	GO:0016020	membrane	3188	245	0.00213
	GO:1990204	oxidoreductase complex	62	11	0.00223
	GO:0009573	chloroplast ribulose bisphosphate carbox...	5	3	0.0026

**Group**	**GO ID**	**Term Annotated**	**Total Transcripts**	**Significant Transcripts**	**P-value**
	GO:0048492	ribulose bisphosphate carboxylase comple...	5	3	0.0026
	GO:0000311	plastid large ribosomal subunit	2	2	0.00437
	GO:0009547	plastid ribosome	2	2	0.00437
	GO:0043234	protein complex	897	78	0.00666
