# Supplementary material for: Transcriptome sequencing analysis of alfalfa reveals CBF genes potentially playing important roles in response to freezing stress
Source: Genet Mol Biol. 2017 Nov 6;40(4):824–33. doi: 10.1590/1678-4685-GMB-2017-0053 (PMC5738619; doi:10.1590/1678-4685-GMB-2017-0053)
Supplement: Supplementary file 5 [file 1415-4757-gmb-1678-4685-GMB-2017-0053-Suppl07.pdf]

Supplementary Material to “Transcriptome sequencing analysis of alfalfa reveals CBF genes potentially playing important roles in response to freezing stress”

Table S3 - Transcripts homologous to AP2/ERF genes differentially expressed in response to cold and/or freezing stress. The transcripts set in bold indicate homology to the CBF cluster in *Medicago truncatula*.

Transcript	Homologue	Chromosome location	Group
MsUN14668	MtERF021	chr1:26538338-26539007	DREB
MsUN35304	MtERF023	chr1:45888187-45888799	DREB
MsUN02379	MtERF071	chr2:4034815-4036032	ERF
MsUN12216	MtERF013	chr3:32669719-32670657	DREB
MsUN02792	MtERF014	chr3:47037850-47039084	DREB
MsUN25615	MtERF054	chr3:49532324-49533111	ERF
MsUN03686	MtERF073	chr4:30411899-30413146	ERF
MsUN29238	MtERF027	chr4:42553246-42554433	DREB
MsUN12932	MtERF028	chr5:1814617-1815198	DREB
MsUN34151	MtERF031	chr5:3037835-3038624	DREB
**MsUN36537**	**MtERF036**	**chr6:23257085-23258239**	**DREB**
**MsUN07888**	**MtERF037**	**chr6:23265303-23267001**	**DREB**
**MsUN28348**	**MtERF039**	**chr6:23289566-23290162**	**DREB**
**MsUN31996**	**MtERF039**	**chr6:23289566-23290162**	**DREB**
**MsUN05203**	**MtERF040**	**chr6:23390649-23391838**	**DREB**
**MsUN05159**	**MtERF042**	**chr6:23586674-23587485**	**DREB**
**MsUN10314**	**MtERF042**	**chr6:23586674-23587485**	**DREB**
**MsUN37647**	**MtERF042**	**chr6:23586674-23587485**	**DREB**
**MsUN36498**	**MtERF043**	**chr6:23633759-23634451**	**DREB**
MsUN06973	MtERF005	chr8:38012580-38014120	DREB
