# Supplementary material for: Transcriptome sequencing analysis of alfalfa reveals CBF genes potentially playing important roles in response to freezing stress
Source: Genet Mol Biol. 2017 Nov 6;40(4):824–33. doi: 10.1590/1678-4685-GMB-2017-0053 (PMC5738619; doi:10.1590/1678-4685-GMB-2017-0053)
Supplement: Supplementary file 7 [file 1415-4757-gmb-1678-4685-GMB-2017-0053-Suppl08.pdf]

Supplementary Material to “Transcriptome sequencing analysis of alfalfa reveals CBF genes potentially playing important roles in response to freezing stress”

Table S4 - Hormone-related transcripts that were differentially expressed response to cold and/or freezing stress.

Hormone	Transcripts	Homologous gene	log_2(fold)		Annotation
			Cold	Freezing	
ABA	MsUN03758	Medtr1g008500	-1.02	-2.23	BURP domain-containing protein
	MsUN03759	Medtr1g008500	-1.37	-2.68	BURP domain-containing protein
	MsUN17391	Medtr2g081770	-0.02	2.96	BURP domain-containing protein
	MsUN20467	Medtr2g030540	-1.14	-0.69	ABA Overly-Sensitive 5
	MsUN29451	Medtr2g081610	-1.65	0.22	BURP domain-containing protein
	MsUN30665	Medtr1g025220	1.32	0.75	GAST1 protein homolog 1
	MsUN31017	Medtr1g025220	1.73	0.78	GAST1 protein homolog 1
	MsUN34141	Medtr7g118290	0.24	-1.36	photosystem I P subunit
	MsUN34420	Medtr4g029350	-1.37	-2.44	lipid transfer protein 3
	MsUN34471	Medtr4g428370	-1.01	-1.65	lipid transfer protein 3
	MsUN34472	Medtr4g428370	-0.86	-2.9	lipid transfer protein 3
	MsUN56879	Medtr1g008500	-1.16	-1.67	BURP domain-containing protein
	MsUN66001	Medtr4g098850	3.21	1.51	myo-inositol polyphosphate 5-phosphatase 2
	MsUN72764	Medtr4g105250	1.19	0.37	Raffinose synthase family protein
	MsUN74926	Medtr8g015480	1.15	0.54	AT3G21320.1
Auxin	MsUN04438	Medtr4g072680	1.36	0.05	SAUR-like auxin-responsive protein family
	MsUN05066	Medtr4g072770	-0.76	-2.44	SAUR-like auxin-responsive protein family

Hormone	Transcripts	Homologous gene	log$_2$(fold)		Annotation
			Cold	Freezing	
	MsUN08547	Medtr4g072190	-1	-0.72	SAUR-like auxin-responsive protein family
	MsUN14170	Medtr3g117610	1.05	0.04	SAUR-like auxin-responsive protein family
	MsUN17771	Medtr1g052885	0.16	1.81	pinoid-binding protein 1
	MsUN21958	Medtr0035s0150	1.51	0.25	Auxin-responsive GH3 family protein
	MsUN22407	Medtr8g011650	-0.66	-2.17	auxin-responsive family protein
	MsUN28358	Medtr4g072980	-0.78	-1.03	SAUR-like auxin-responsive protein family
	MsUN30914	Medtr4g072730	-0.29	-1.28	SAUR-like auxin-responsive protein family
	MsUN31249	Medtr2g008050	0	1.64	actin-11
	MsUN43181	Medtr4g072500	-0.47	-1.11	SAUR-like auxin-responsive protein family
	MsUN55649	Medtr4g072370	1.29	0.53	SAUR-like auxin-responsive protein family
	MsUN62814	Medtr0388s0020	0.51	-1.26	SAUR-like auxin-responsive protein family
GA	MsUN14333	Medtr4g106860	1.35	3.46	Gibberellin-regulated family protein
	MsUN14971	Medtr3g102660	-0.7	-1.07	Gibberellin-regulated family protein
	MsUN21671	Medtr1g018640	1.27	-0.18	Gibberellin-regulated family protein
	MsUN21705	Medtr3g037750	-1.08	-1.61	Gibberellin-regulated family protein
	MsUN26349	Medtr4g081950	-1.05	-0.56	expansin A1
	MsUN27237	Medtr7g090590	-1.09	-2.78	Gibberellin-regulated family protein
	MsUN34780	Medtr7g010580	-1.56	-1.36	GAST1 protein homolog 4
	MsUN41086	Medtr4g076140	-1	-0.41	scarecrow-like 3
BA	MsUN07796	Medtr5g064360	1.6	2.84	Phosphate-responsive 1 family protein
	MsUN29316	Medtr3g074830	1.9	3.51	Phosphate-responsive 1 family protein
	MsUN30876	Medtr3g074860	2.69	1.7	Phosphate-responsive 1 family protein
	MsUN31693	Medtr5g064360	1.72	2.32	Phosphate-responsive 1 family protein
JA	MsUN03654	Medtr5g013530	2.83	1.1	jasmonate-zim-domain protein 1
	MsUN04425	Medtr2g042900	3.17	1.18	jasmonate-zim-domain protein 1
	MsUN21768	Medtr8g021380	2.42	0.76	jasmonate-zim-domain protein 6
	MsUN34353	Medtr4g124960	1.69	0.22	jasmonate-zim-domain protein 1

Hormone	Transcripts	Homologous gene	log_2(fold)		Annotation
			Cold	Freezing	
Others	MsUN02075	Medtr3g064050	0.49	1.19	auxin response factor 8
	MsUN09064	Medtr4g082860	0.7	-1.3	nodulin-related protein 1
	MsUN22344	Medtr4g083110	1.58	-1.16	AT1G30260.1
	MsUN25660	Medtr3g100620	-0.97	-1.37	G-box regulating factor 6
	MsUN65110	Medtr7g062540	0.84	2.51	auxin response factor 18
