# Supplementary material for: Transcriptome sequencing analysis of alfalfa reveals CBF genes potentially playing important roles in response to freezing stress
Source: Genet Mol Biol. 2017 Nov 6;40(4):824–33. doi: 10.1590/1678-4685-GMB-2017-0053 (PMC5738619; doi:10.1590/1678-4685-GMB-2017-0053)

Supplementary Material to "Transcriptome sequencing analysis of alfalfa reveals CBF genes potentially playing important roles in response to freezing stress"

Figure S4 - Expression models of four JAZ genes in response to cold and/or freezing stress in *Medicago sativa* and *M. truncatula*.

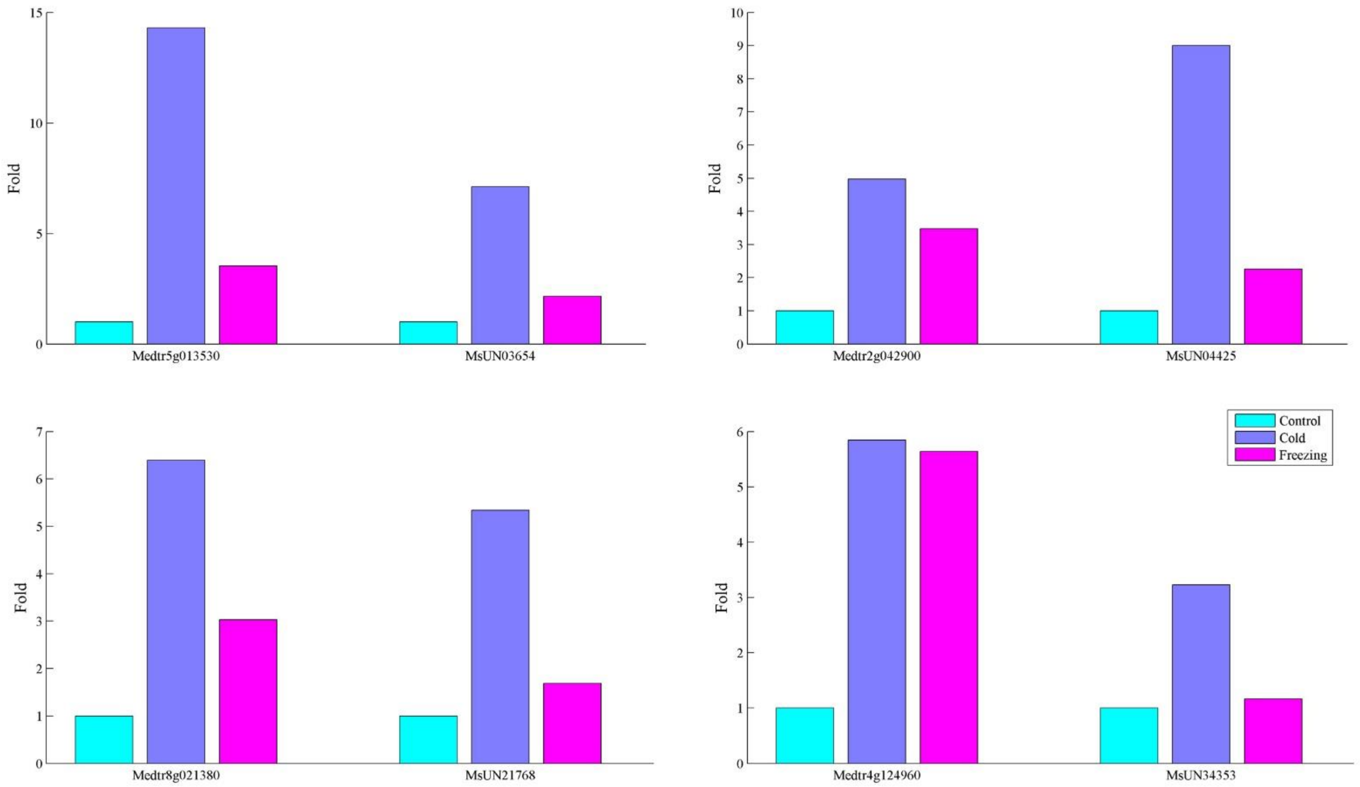

Supplement: Supplementary file 8 [file 1415-4757-gmb-1678-4685-GMB-2017-0053-Suppl04.pdf]
